# Using a Secure, Continually Updating, Web Source Processing Pipeline to Support the Real-Time Data Synthesis and Analysis of Scientific Literature: Development and Validation Study

**DOI:** 10.2196/25714

**Published:** 2021-05-06

**Authors:** Uddhav Vaghela, Simon Rabinowicz, Paris Bratsos, Guy Martin, Epameinondas Fritzilas, Sheraz Markar, Sanjay Purkayastha, Karl Stringer, Harshdeep Singh, Charlie Llewellyn, Debabrata Dutta, Jonathan M Clarke, Matthew Howard, PanSurg REDASA Curators, Ovidiu Serban, James Kinross

**Affiliations:** 1 PanSurg Collaborative Department of Surgery and Cancer Imperial College London London United Kingdom; 2 Amazon Web Services UK Limited London United Kingdom; 3 MirrorWeb Limited Manchester United Kingdom; 4 Cloudwick Technologies Newark, CA United States; 5 see Acknowledgments London United Kingdom; 6 Data Science Institute Imperial College London London United Kingdom

**Keywords:** structured data synthesis, data science, critical analysis, web crawl data, pipeline, database, literature, research, COVID-19, infodemic, decision making, data, data synthesis, misinformation, infrastructure, methodology

## Abstract

**Background:**

The scale and quality of the global scientific response to the COVID-19 pandemic have unquestionably saved lives. However, the COVID-19 pandemic has also triggered an unprecedented “infodemic”; the velocity and volume of data production have overwhelmed many key stakeholders such as clinicians and policy makers, as they have been unable to process structured and unstructured data for evidence-based decision making. Solutions that aim to alleviate this data synthesis–related challenge are unable to capture heterogeneous web data in real time for the production of concomitant answers and are not based on the high-quality information in responses to a free-text query.

**Objective:**

The main objective of this project is to build a generic, real-time, continuously updating curation platform that can support the data synthesis and analysis of a scientific literature framework. Our secondary objective is to validate this platform and the curation methodology for COVID-19–related medical literature by expanding the COVID-19 Open Research Dataset via the addition of new, unstructured data.

**Methods:**

To create an infrastructure that addresses our objectives, the PanSurg Collaborative at Imperial College London has developed a unique data pipeline based on a web crawler extraction methodology. This data pipeline uses a novel curation methodology that adopts a human-in-the-loop approach for the characterization of quality, relevance, and key evidence across a range of scientific literature sources.

**Results:**

REDASA (Realtime Data Synthesis and Analysis) is now one of the world’s largest and most up-to-date sources of COVID-19–related evidence; it consists of 104,000 documents. By capturing curators’ critical appraisal methodologies through the discrete labeling and rating of information, REDASA rapidly developed a foundational, pooled, data science data set of over 1400 articles in under 2 weeks. These articles provide COVID-19–related information and represent around 10% of all papers about COVID-19.

**Conclusions:**

This data set can act as ground truth for the future implementation of a live, automated systematic review. The three benefits of REDASA’s design are as follows: (1) it adopts a user-friendly, human-in-the-loop methodology by embedding an efficient, user-friendly curation platform into a natural language processing search engine; (2) it provides a curated data set in the JavaScript Object Notation format for experienced academic reviewers’ critical appraisal choices and decision-making methodologies; and (3) due to the wide scope and depth of its web crawling method, REDASA has already captured one of the world’s largest COVID-19–related data corpora for searches and curation.

## Introduction

Between December 31, 2019, and August 3, 2020, 37,362 papers related to COVID-19 were published on PubMed [1], with Dimensions reporting over 100,743 publications, 1503 policy documents, and 1097 data sets [2]. The speed and scale of the production of published data on COVID-19 across both peer- and nonpeer-reviewed literature presents considerable challenges for stakeholders (eg, policy makers, clinicians, and patients) who must make subjective quality judgements on new data and rapidly synthesize information in order to make optimal, evidence-based decisions. Traditional approaches to data synthesis are unable to keep pace with the rapidly changing information landscape. For example, in the United Kingdom, the National Institute for Health and Care Excellence was unable to publish their initial therapeutic guidance on managing COVID-19 until March 20, 2019 [3]. Ultimately, they modified their methodology for publishing rapid guidance materials on COVID-19 [4]. Moreover, there have been concerns regarding data credibility and the political misuse of information, resulting in the World Health Organization announcing its campaign for discouraging the spread of misinformation [5]. The COVID-19 pandemic highlights the urgent need to prospectively capture, structure, and interpret expansive and complex data sets in real time to support the rapid development of clinical guidance and, most critically, ensure that various key stakeholders can make the best possible evidence-based decisions during an “infodemic.”

Previous strategies have attempted to address these challenges by using the concepts of live systematic reviews, which involve the continuous, structured analysis of data that target specific clinical questions [6,7] as well as the clear presentation of such data [8]. However, despite the progress in this field, major obstacles remain in establishing automated data mining frequency, depth, and robustness. Moreover, major barriers exist in the development and validation of machine learning methodologies for the autonomous analysis of heterogeneous clinical data sets.

This paper outlines the methodology of REDASA (Realtime Data Synthesis and Analysis)—a novel, prospective clinical information platform that was developed during the COVID-19 pandemic. It was designed for use across a wide range of data-rich subject areas while keeping application and impact in mind. Our objective was to continuously capture and synthesize both academic and relevant grey literature (eg, news websites, policy documentation, and social media posts) on COVID-19 and to develop a validated data curation approach that could supplement machine learning methodologies and be used as the basis for conducting live systematic reviews.

## Methods

### Components of REDASA

REDASA was built and deployed on the Amazon Web Service (AWS) cloud. Cloud computing is the on-demand delivery of compute power, database storage, applications, and other information technology resources through a cloud service platform on the internet. A cloud services platform, such as AWS, owns and maintains the network-connected hardware required for these application services. By using the AWS cloud, REDASA components were rapidly designed. REDASA components were integrated and deployed by using AWS tools and the solutions developed by AWS Partners, MirrorWeb, and Cloudwick. These components were comprised of a real-time data extraction pipeline that was implemented by using MirrorWeb’s digital archiving technology, a data lake storage repository and workflow orchestration platform (Amorphic) that was developed by Cloudwick, a natural language search engine that was implemented by using Amazon Kendra, and a document curation pathway that was implemented by using Amazon SageMaker Ground Truth.

### Real-Time Data Extraction Pipeline

MirrorWeb was used to conduct an exploratory review of the target websites via manual and automatic content detection for informing crawl scoping decisions. Exploratory reviews involve the domain composition analysis of initial web estate archives, which can be produced via multiple methods, including basic link harvesting, Domain Name System lookups, the gathering of URL lists at crawl time (to identify content delivery networks and perform manual verifications), and the inspection of websites. This ensures that (1) the relevant areas of websites are identified and followed by the archive tools and (2) content that can be confidently omitted is avoided. With the adoption of machine learning algorithms, this process can be further assisted by technology.

Scoping decisions can encompass a range of factors. For instance, scoping decisions can ensure that the website crawl stays within the target domain. Further, they can refine the website crawl to only include relevant URL patterns within the domain. For example, if there is a domain.com/coronavirus/ subfolder that contains relevant content, the web crawler will use a series of URL pattern matches and regular expressions to allow or disallow URL strings via exact matching or wildcard pattern matching, thereby containing the crawl to specific areas of the website. Additionally, scoping decisions can expand the crawl scope to include any outlying content that would be excluded by the refinement rules. Some websites have nonstandard locations for storing assets such as images, stylesheets, and scripts, which are needed to create a high-fidelity representation of the source material. Some websites also contain relevant documents that are located in prescribed location structures outside of the primary target folder.

Successful, high-fidelity targeted web crawling has been well documented. However, considerable challenges remain in the development of a qualitative and quantitative method for real-time relevant URL detection [9]. This is because the web ecosystem is a constantly evolving landscape with continuous advancements in available technology; construction techniques; and the consideration of desktop, mobile, and accessible display devices. Furthermore, the sheer number of content management systems that adopt their own proprietary content structures, the advent of single page applications, the prevalence of JavaScript, and people’s dependence on asynchronous loading and POST method requests (which returns the same URL for multiple requests) render traditional URL similarity detection a particular challenge. Programmatic links with no human-readable semantic structures and features, such as “Latest News” sections within a web page, can often skew the results of link-based page relevance analyses.

These challenges are exacerbated in the REDASA system, which is required to target data in both academic and nonacademic sources without a guaranteed schema, dialect, or topic. Previously developed methodologies for addressing this issue have been used to apply anchor text similarity scores, content similarity scores, and URL prediction scores (which are based on a set of starting keywords) to seed data [10,11]. These scoring models promote and remove keywords based on the detection of commonalities in discovered crawl data. However, this approach presents several challenges because it relies on good starting URLs that present a reliable and consistent pattern of data. To counter this, REDASA performs downstream content filtering after the crawl is complete in order to eliminate extraneous data, which eliminates the risk of losing vital information that comes with the analysis of a potentially biased set of keywords. Due to REDASA’s ability to perform a retrospective analysis of retained data, it will serve as a future platform that can be further enhanced by discovery automation.

**Figure 1:**
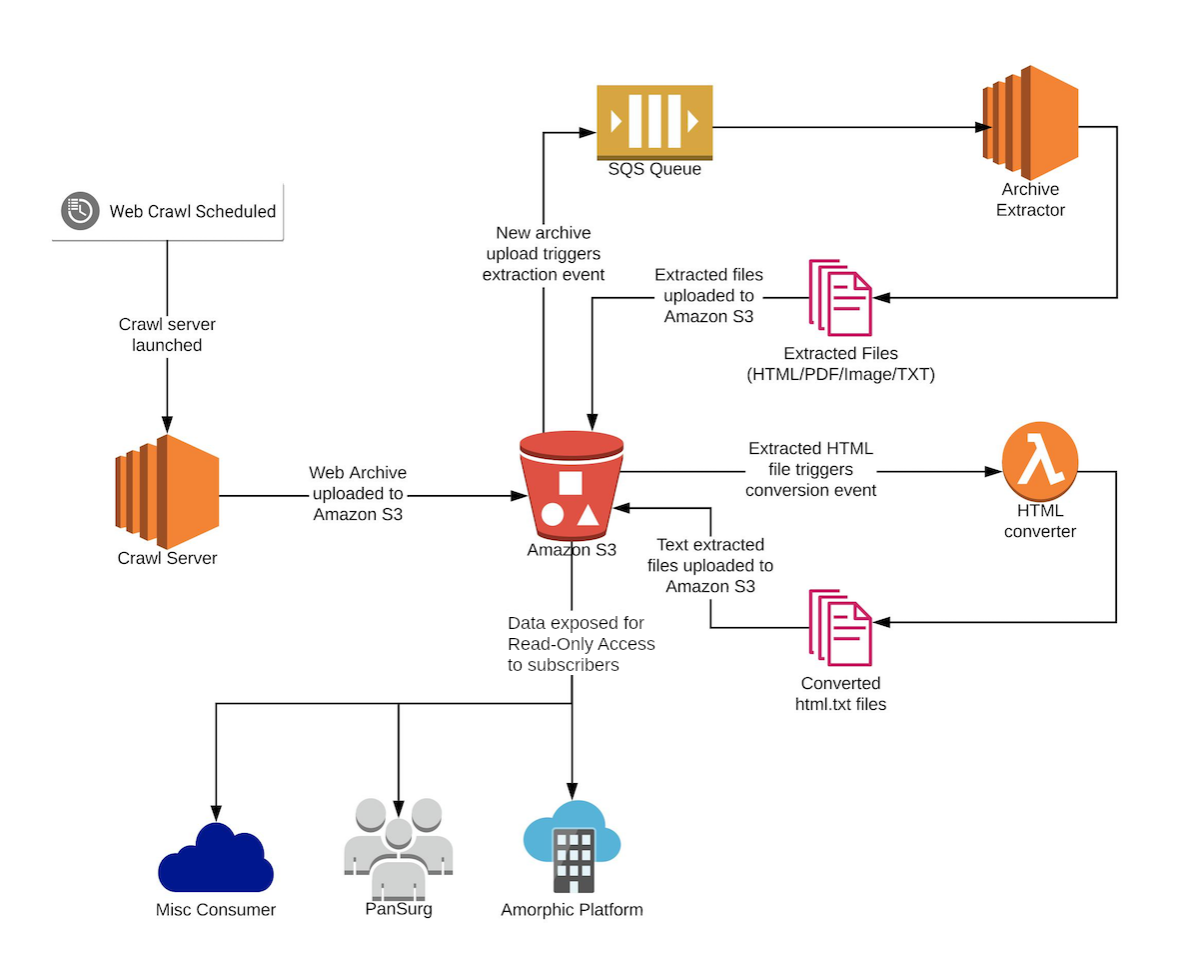
The REDASA back-end web crawling and data processing pipeline. REDASA: Realtime Data Synthesis and Analysis; SQS: Simple Queue Service; TXT: text.

In practice, REDASA performs an initial crawl that is launched by using crawl scope definitions that are governed by the aforementioned decision rules. The process for completing a crawl is outlined in Figure 1, which provides an interactive replica of the website content that is accessible to curators. MirrorWeb’s Crawl Quality team reviewed the quality of the archive by using a replay engine to create a navigable replica of the target website archive. In addition to clicking the links and manually reviewing the content, an automated link checker was used to recursively spider the archive, identify page and document links in the HTML content, and attempt to open the target URLs in the archive instance in an effort to detect any missing referenced content. If any changes to the scoping rules are needed to make the web crawl more permissive or more restrictive, the scoping rules will be amended as required by using the same, aforementioned scoping principles. This iterative cycle repeats until the crawl is of sufficient quality to be used for human curation and accurate natural language processing (NLP) searches.

### Data Lake

Cloudwick’s Amorphic platform provides a core REDASA data lake repository and data workflow orchestration service. MirrorWeb data initially lands in the storage layer of Amorphic, which consists of a landing zone. After validation checks are performed, data are moved and stored in a data lake zone and made available for document curation and search index workflows, as described in the following section.

### Search Index for Question-Specific Curation Documents

**Figure 2:**
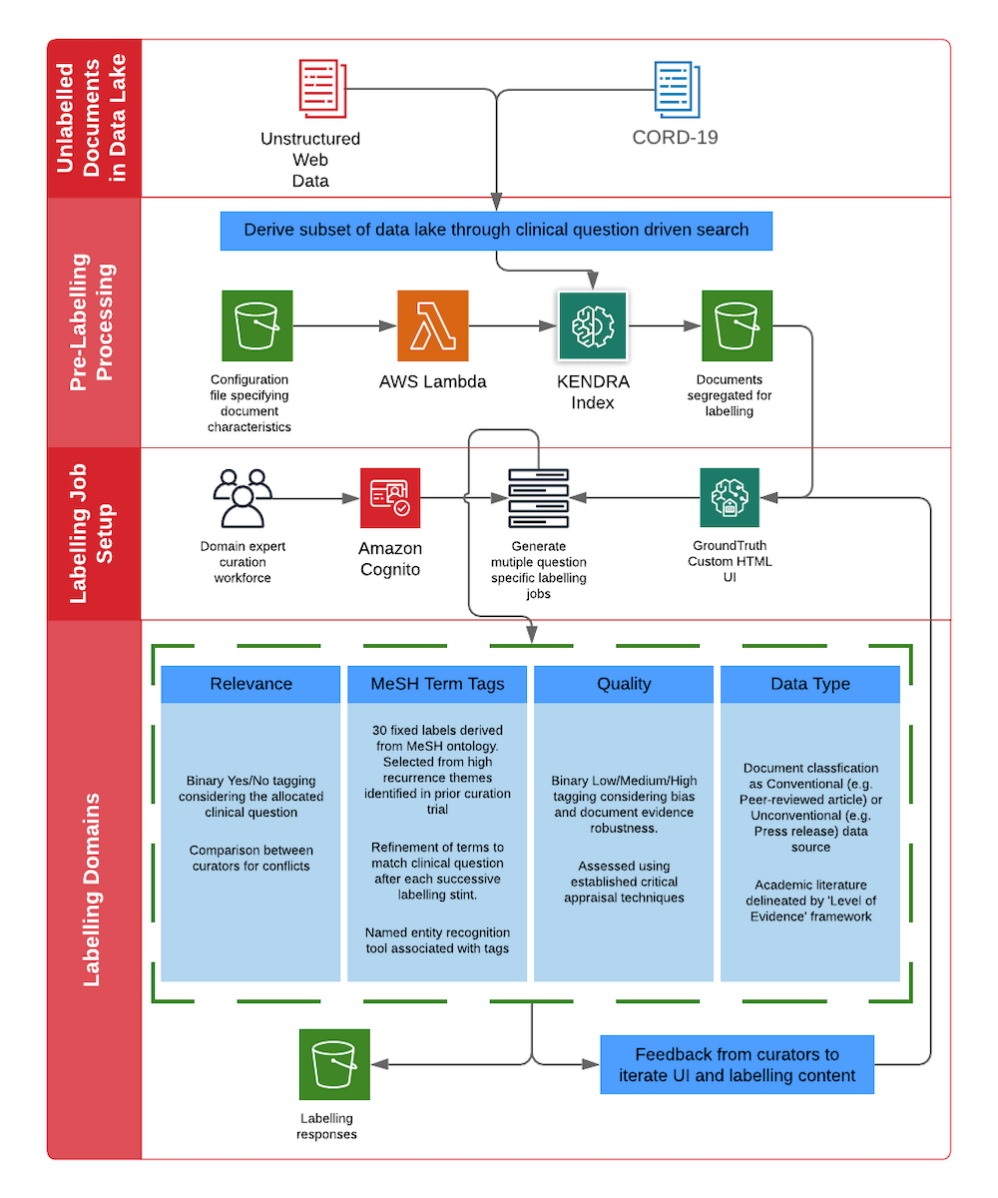
Integrated workflow of the search index and data curation pipeline for a variety of high-impact areas with and without consensus among the scientific community in different countries and health authority bodies. AWS: Amazon Web Service; CORD-19: COVID-19 Open Research Dataset; MeSH: Medical Subject Headings; UI: user interface.

REDASA uses an AWS enterprise search service—Amazon Kendra—to provide search functionality across the entire data lake. Amazon Kendra is an NLP machine learning service that uses deep learning models to understand natural language queries and document content and structures. Amazon Kendra provides support for the following three broad types of questions: (1) factoid questions (who, what, when, and where), which are questions that require fact-based answers that may be returned in the form of a single word or phrase (the precise answer however must be explicitly stated in the ingested text content); (2) descriptive questions, which involves answers that could be a sentence, passage, or an entire document; and (3) keyword searches, wherein the intent and scope of the question may not be clear. In REDASA’s question-specific curation model, Amazon Kendra exclusively received factoid questions and used a series of deep learning models to return relevant documents (Figure 2).

The key component of Amazon Kendra is an index. Conceptually, an index is an abstraction that encompasses a set of documents and the underlying hardware and software infrastructure that makes it possible to query documents that use natural language. Aside from its actual content, each document may include some associated metadata (eg, the source of the document, the document’s logical unit group, etc). Users can specify custom metadata fields to suit their needs. These metadata tags are accessible through the Amazon Kendra query application programming interface.

A Kendra index may consist of one or more data sources, and a data source is a logical unit of documents. For REDASA, data source file types were limited to plain text, HTML, and PDF. Compared to other file types, these better integrate with our curation platform and allow for consistent labeling outputs when performing named entity recognition (NER).

### Document Curation

Document curation was implemented by using the custom workflows in Amazon SageMaker Ground Truth, which is a data labeling service that is used to build training data sets for machine learning workflows. REDASA uses a question-based curation approach. PanSurg investigators posed a series of COVID-19–related key questions to the search index (Textbox 1). These questions were chosen to obtain answers, and we were able to validate the quality of the data lake and the adequacy of REDASA’s data mining depth with our curation relevance metric.

**COVID-19–related natural language processing queries that were posed to the REDASA (Realtime Data Synthesis and Analysis) search index to develop a question-specific curation methodology.**

**Queries in natural language**

1. What is the time interval between SARS-CoV-2 infection and testing positive?
2. What is the most sensitive imaging modality to diagnose COVID-19?
3. Which underlying health conditions increase mortality?
4. What is the risk of SARS-CoV-2 infection in health care professionals?
5. Should laparoscopy be performed on SARS-CoV-2 positive patients?
6. What is the estimated global economic impact of COVID-19?
7. How effective are masks at minimizing the transmission of COVID-19 in public?
8. What is the evidence for COVID-19 mutations and how many subtypes are responsible for the pandemic?
9. Does a positive SARS-CoV-2 antibody test mean an individual is immune to further COVID-19 infections?
10. Is COVID-19 airborne transmitted?
11. Can asymptomatically infected individuals transmit COVID-19?
12. What is the evidence for 1-meter and 2-meter separations for social distancing?
13. What has the evidence-base been for lockdown compared to no lockdown during this COVID-19 pandemic?
14. Is universal preoperative testing for SARS-CoV-2 beneficial compared to selective testing?
15. Can individuals be reinfected with SARS-CoV-2?

**Figure 3:**
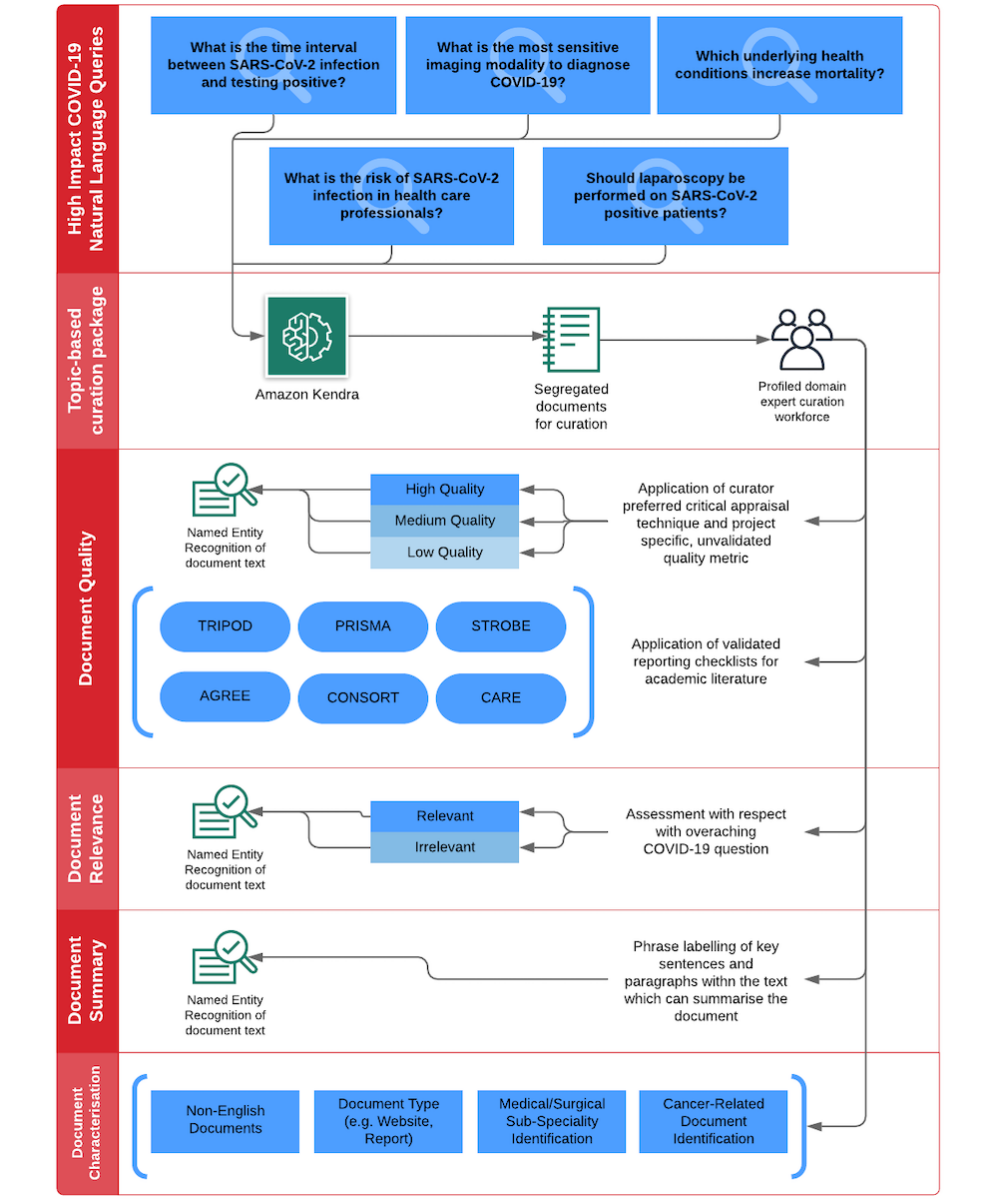
Curation labels for generating document metadata. AGREE: Appraisal of Guidelines for Research and Evaluation; CARE: Case Reports; CONSORT: Consolidated Standards of Reporting Trials; PRISMA: Preferred Reporting Items for Systematic Reviews and Meta-Analyses; STROBE: Strengthening the Reporting of Observational Studies in Epidemiology; TRIPOD: Transparent Reporting of a Multivariable Prediction Model for Individual Prognosis or Diagnosis.

The REDASA search index provides a list of selected documents, which are randomly provisioned to curators for labeling via Amazon SageMaker Ground Truth. This allows them to assess documents’ relevance and quality in relation to the original query and further categorize the data based on the labels described in Figure 3. Reflecting the living nature of the REDASA platform, queries were adapted in accordance with the knowledge priorities of different phases of the pandemic. For example, in stint 1 of curation (February 6 to September 6, 2020), which was scheduled during the peak of the UK COVID-19 outbreak and when uncertainties regarding best practices for screening and management planning were rife, questions 1-5 were posed to REDASA. In contrast, stint 2 of curation was performed during the nationwide lockdown relaxation period and the public health transition for minimizing the risk of a second wave of COVID-19. Consequently, questions 6-15 focused upon themes such as reinfection, transmission mitigation, and the global impact of the pandemic.

The relevance of articles in relation to the query that was posed to the search index was a subjective binary measure (ie, irrelevant or relevant). This assessment was paired with NER labels, which enabled curators to highlight phrases and paragraphs that indicated the relevance of articles.

**Figure 4:**
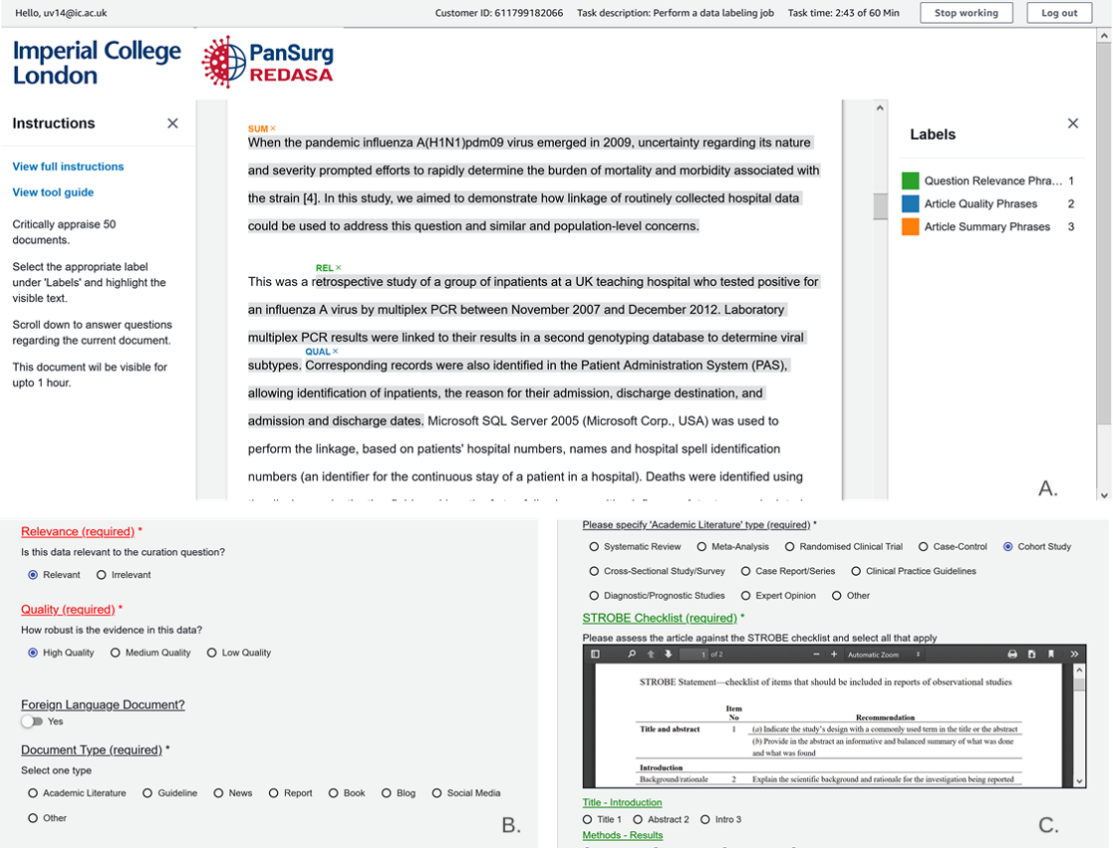
(A) A document with curation user interface labels (the NER of quality, relevance, and summary phrases). (B) Binary labels for classifying documents and correlating them to NER responses. (C) Embedded reporting checklists for document assessment, which were provided based on the selected academic study type. NER: named entity recognition; REDASA: Realtime Data Synthesis and Analysis; STROBE: Strengthening the Reporting of Observational Studies in Epidemiology.

The quality of the academic literature was assessed via a 3-stage process. First, we ascertained the study type, and this allowed us to assign an evidence rating level (the levels proposed by the Oxford Centre for Evidence-Based Medicine [12]). Second, we invited curators to provide an independent, subjective rating of an article’s quality by using their own critical appraisal methodology and assign 1 of the 3 following binary ratings: low, medium, or high. Third, akin to the relevance metric, NER annotation was made available to curators and correlated with their low, medium, or high ratings. Depending on the type of academic literature that curators were assessing, curators were automatically given (through their user interface) the relevant EQUATOR (Enhancing the Quality and Transparency of Health Research) checklist for quantitative quality assessment [13]. For example, if the document that curators were assessing was a systematic review, they were automatically able to assess the article against the PRISMA (Preferred Reporting Items for Systematic Reviews and Meta-Analyses) checklist (Figure 4).

Collectively, the relevance and quality metrics’ utility was threefold. First, they enabled us to capture data on curators’ decision-making and critical appraisal processes. Second, they minimized the number of undesirable irrelevant documents, which allowed us to implement a human-in-the-loop optimization methodology for the search index. Third, they allowed us to perform multiple curator passes on a single document, assess for labeling response conflicts, and ascertain the article factors responsible for any disparities. To obtain further data, we allowed our curators to assess the risk of bias in the articles by using the bias metrics designed by Cochrane [14]. The results from this novel curation process were intended to (1) act as ground truth for data science models that aimed to facilitate the future semiautomation or full automation of article screening, and (2) be used for a structured assessment of evidence quality.

This question-specific approach was selected over the more traditional approach of randomly sectioning data to help us preserve the relevance metric for specific questions. This factor would have otherwise been more challenging to implement and capture. Further, this metric is key to future work streams that determine the relevance of specific articles for inclusion in an automated systematic review.

### Curation Methodology

#### Structured and Unstructured Data Lake

Our proof-of-concept analysis for data mining during predefined time periods was feasible. In this iteration of REDASA, a 1-week time period was chosen to enable the capturing of the highest possible number of new data points at the lowest mining frequency, thus minimizing the computing costs of both COVID-19–structured and unstructured data sources. In this paper, we only present a text-based analysis of the data set. In the future, we intend to assess structured, quantitative data from target data sources.

#### Unstructured Web Crawl Data

Textual information was extracted from a precurated set of internet sites. A range of frequently accessed but disparate data types that are typically used by frontline clinicians and policy makers were extracted. These included high-quality journal websites and portals containing COVID-19–related literature; medical and surgical society guidance web pages; and guideline repositories from local, governmental, and international public health bodies. By being able to dynamically capture data and automatically obtain updates from sources, this type of data mining demonstrates the power of REDASA in terms of amalgamating qualitative and quantitative insights for generating future reports. Each website was independently assessed and evaluated for inclusion into the REDASA data lake by clinical (n=4) and data science (n=2) reviewers. Disagreements were resolved through consensus. These sources were selected in accordance with criteria for including usable content and determining the reliability and breadth of target topics and categories pertaining to COVID-19. To systematize the data lake prior to data ingestion, sources were categorized into the following broad groups, which were independently defined a priori by the three members of the research team based on the source of the original data: all (miscellaneous), critical care, medical care, surgical care, drug development and pharmacological therapy, mental health, risk, translational research, biological sciences, engineering, and policy. The content of the data from each of the sources was screened by these three independent members of the research team. If disagreements regarding categorization occurred, a meeting was conducted. Unanimous agreement was sought prior to final categorization.

#### Structured Data From the COVID-19 Open Research Dataset

The White House and a coalition of leading research groups developed the COVID-19 Open Research Dataset (CORD-19) [15]. The CORD-19 is a data set that contains over 157,000 scholarly articles, including over 75,000 full-text articles regarding COVID-19, SARS-CoV-2, and related coronaviruses. This freely available data set was provided to the global research community of Kaggle and was used as a test data source when initially developing the REDASA infrastructure.

Collectively, these assimilated data sources make REDASA one of the world’s largest and most contemporaneous COVID-19–related evidence sources, consisting of 104,000 documents.

### Data Availability

The curation labels can be found on GitHub [16].

## Results

### Curation Results

The first-pass document curation responses from 42 curators underwent the preliminary analysis of the domains of relevance to free-text queries and information quality. These data were collected over 2 time-limited curation stints. Both stints were 1 week in duration. We obtained a total of 1424 documents pertaining to 15 different COVID-19–related queries (around 99 text documents derived per query from the search index for the structured and unstructured data lake), and an average of 42 documents per query were assessed by 1 curator. Our aim was for each document to be assessed by at least 2 different reviewers so that we could assess intercurator variability and identify the reasons for discrepancies in evidence quality verdicts.

**Figure 5:**
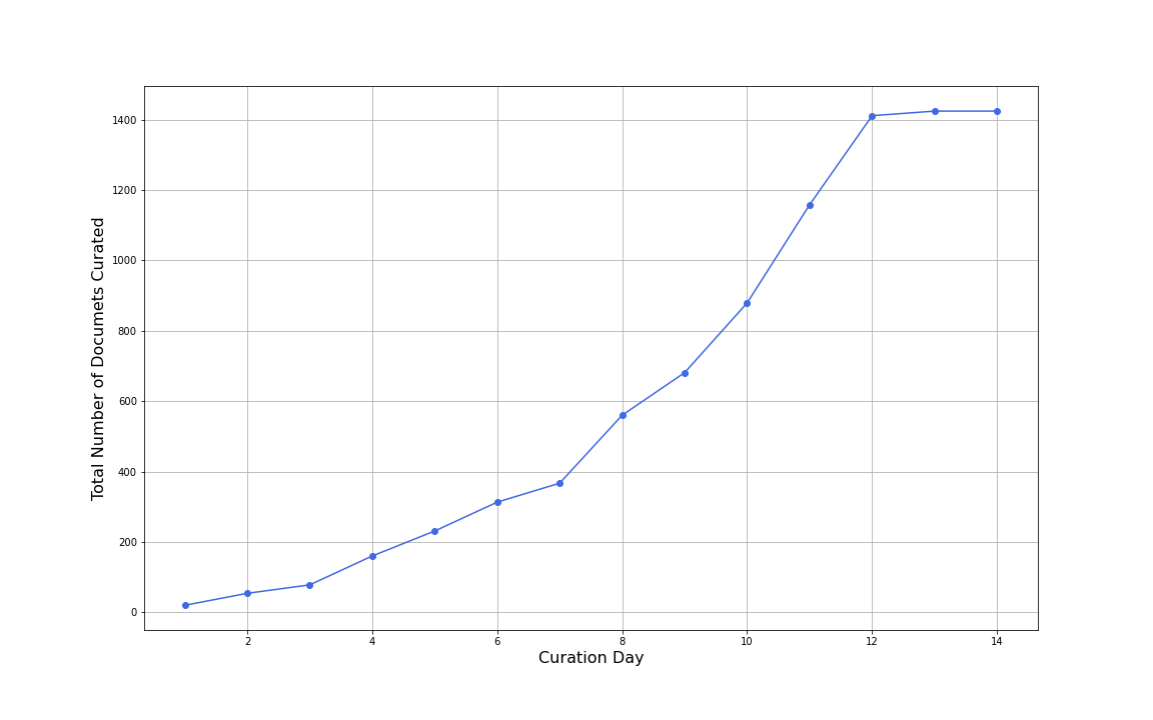
Rate of COVID-19–related scientific literature curation over 2 weeks. This was associated with the growth of the number of curators, which plateaued on day 13. This was when all of the documents available for curation were assessed before the end of stint 2.

Each curator was profiled to ascertain their academic or clinical backgrounds. This was initially performed for the vetting and quality control of curation responses. These data will also be used to further discriminate between labeling responses that are based on critical appraisal expertise and to assign weights to curation responses. To date, the REDASA project’s international curator community includes people from 9 different countries, and the project is supported by medical and surgical health care professionals who range from senior consultant vascular surgeons in Italy to general practice physicians who are involved in community and public health decision making in the Philippines. Data curators were recruited by invitation through the PanSurg email subscriber list and by open invitation via the @pansurg Twitter account. Curators were included if they had a medical degree or a higher degree in science-related fields. Data curators were asked to state their interest and were verified by direct contact. The number of data curation responses for REDASA, which exponentially rose between our two stints as more curators were onboarded (stint 1: n=12; stint 2: n=42; Figure 5), was indicative of an efficient, novel methodology for the digital, community-based peer review of literature by domain experts. This was exemplified by the ability of some of our experienced curators, who were able review over 100 scientific documents of varying length in as little as 3 days. Furthermore, with curators’ 100% follow-through rate between stint 1 and stint 2, our curation model suggests that, when combined with our simplified critical appraisal interface, the peer review of literature at scale is viable and sustainable.

In total, 70.9% (1009/1424) of the pool of curated articles was composed of peer-reviewed, traditional, academic literature; the remainder consisted of web crawl–derived data, including governmental policies and reports from professional bodies. Based on the subset of the 900 academic literature documents that were curated, the most common study type encountered was systematic reviews (98/1009, 9.7%). The least common study type in the data lake was randomized controlled trials (RCTs; 3/1009, 0.3%). Nonsystematic reviews (eg, rapid reviews and comprehensive reviews) were not given the systematic review label to avoid inappropriate assessments against the PRISMA checklist. Such outlier academic literature types were aggregated into the miscellaneous category, which included 427 documents that were assessed solely against the curator-reported binary quality ratings.

### Articles’ Relevance to Queries

**Figure 6:**
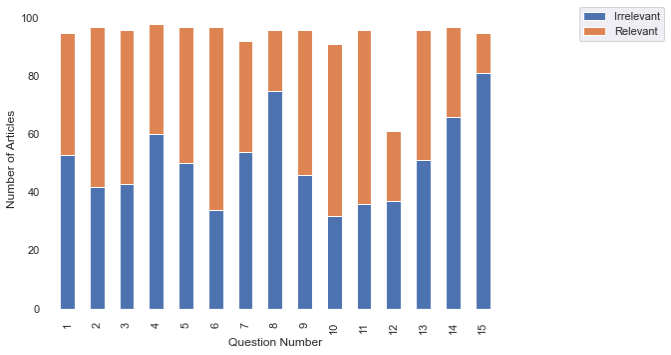
Curators’ responses determined the relevance of documents to search index queries. Responses were matched to the query number.

The relevance metric that curators used provided insight into the performance of the search index in terms of providing cogent and useful document results associated with the 15 COVID-19–related queries (Textbox 1). Overall, 50.49% (719/1424) of articles were considered relevant to their respective queries. When observing the question bank, this variance in article relevance (which was based on the search index) was reflected by the lack of consistency in the ratio of the number of relevant articles to the number of irrelevant articles (Figure 6). These data can be used to provide feedback for the search index with regard to the optimization of provided results.

### Critical Appraisal of Article Quality

The uneven distribution of academic study types that have been curated thus far precludes the interpretation of results for quantitative reporting checklist responses based on the qualitative rating system (low, medium, and high) for RCTs (CONSORT [Consolidated Standards of Reporting Trials] checklist) and clinical guidelines (AGREE [Appraisal of Guidelines for Research and Evaluation] checklist). These studies were poorly represented in this run of analysis.

**Figure 7:**
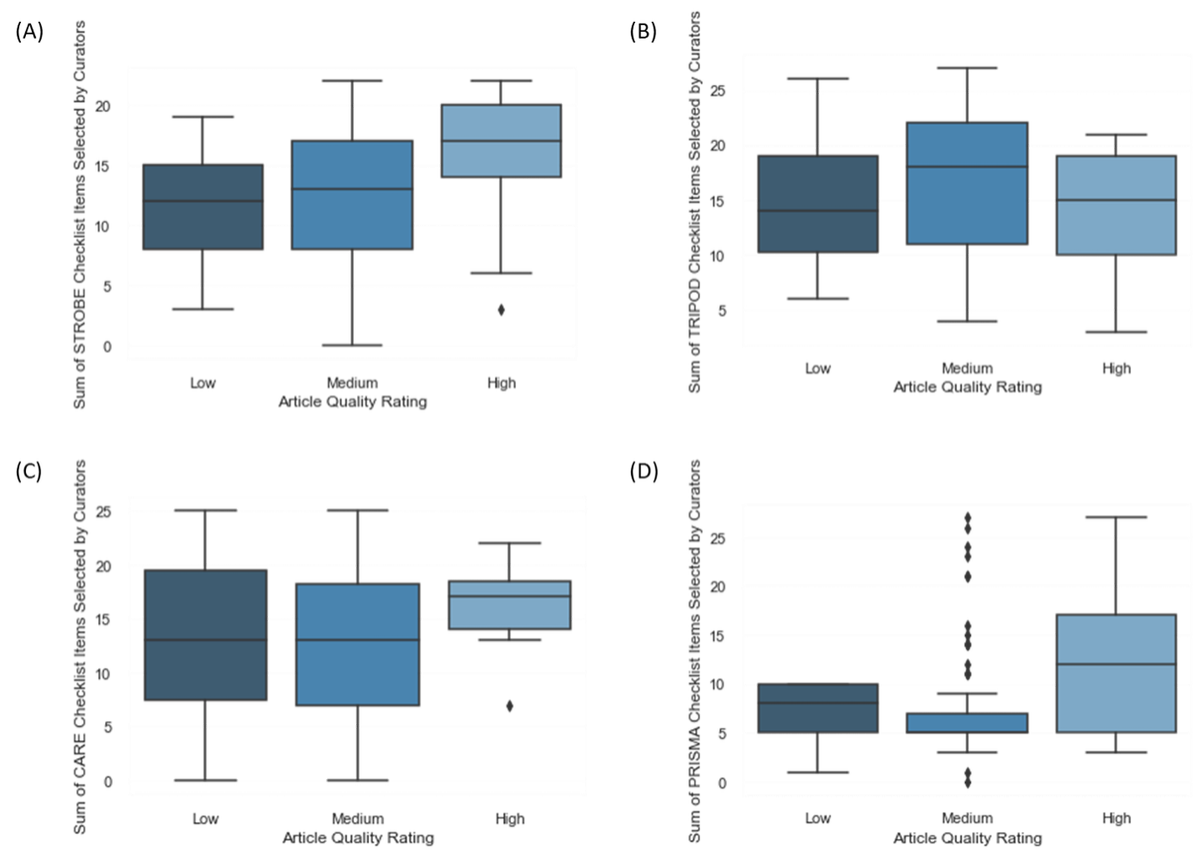
Relationship between the low, medium and, high curator-determined quality ratings of (A) case-control studies, (B) diagnostic and prognostic studies, (C) case reports and series, and (D) meta-analyses and systematic reviews and their respective reporting checklist scores. CARE: Case Reports; PRISMA: Preferred Reporting Items for Systematic Reviews and Meta-Analyses; STROBE: Strengthening the Reporting of Observational Studies in Epidemiology; TRIPOD: Transparent Reporting of a Multivariable Prediction Model for Individual Prognosis or Diagnosis.

Quality was quantified by ascertaining the sum of the number of EQUATOR Network–derived checklist items that were fulfilled by each document. Hence, documents with methods and results that aligned more closely to their respective reporting checklist were scored higher and deemed to be of greater quality. This outcome was compared to the curators’ subjective ratings for diagnostic and prognostic studies (TRIPOD [Transparent Reporting of a Multivariable Prediction Model for Individual Prognosis or Diagnosis] checklist), case reports and series (CARE [Case Reports] checklist), case-control studies (STROBE [Strengthening the Reporting of Observational Studies in Epidemiology] checklist) and meta-analyses and systematic reviews (PRISMA checklist) (Figure 7). Notably, the subjective quality rating was assigned prior to assessment by using the checklist under our curation protocol to mitigate observer bias.

Based on our independently assessed and subjective quality metric, our preliminary results suggested that more than 50% (726/1424, 50.98%) of the documents derived from the REDASA data pipeline were of medium quality, and 13.55% (193/1424) were deemed high quality. Thus, during data aggregation, 64.53% (919/1424) of the documents derived from our data lake for curation were of a sufficient quality to inform their professional decision-making processes or could be used as reliable sources of information.

With regard to the TRIPOD-relevant (score: mean 15.6, SD 6.9) and STROBE-relevant (score: mean 13.2, SD 5.8) study types, there was some correlation between curators’ subjective assessments of the articles that had low-to-high ratings and underwent the validated checklist–based quality assessment. However, with regard to the CARE-relevant (score: mean 13.8, SD 6.8) and PRISMA-relevant (score: mean 8.4, SD 6.1) study types, there was substantial variance in the number of checklist items that were selected for each quality rating, thus indicating an apparent dissociation between these two metrics. Further data collection and the comparison of intercurator responses for the removal of outliers will provide clarity on the role of subjective quality ratings versus the role of validated reporting checklists as a surrogate marker of evidence quality.

## Discussion

Globally, there are several efforts underway for systematically accruing COVID-19–related data and specifically querying these data and output-relevant literature [17]. These efforts, such as Google’s COVID-19 Research Explorer [18], COVIDask [19], and COVIDScholar [20], are fundamentally based on NLP searches. Additionally, Google’s solution incorporates the use of follow-up queries associated with a primary question to obtain more focused results. These efforts universally incorporate the CORD-19 and intuitively present output data. Nevertheless, these approaches do not account for the quality of the data source, which is left to the interpretation of the user. Other efforts, such as SciSight [21] and COVID-19 Primer [22], structure data from the CORD-19 into themes (author and engagement), thereby allowing users to make links and review specific topics, albeit without a natural language interface for answering specific questions.

The crucial difference between REDASA and the aforementioned platforms is threefold. First, REDASA adopts a human-in-the-loop methodology by embedding an efficient, user-friendly curation platform into an NLP search engine. REDASA can iteratively refine its search outputs at scale, particularly in the domains of the relevance and quality of data sources. This can ultimately contribute to a fact-checking function for conducting a reliable assessment of the utility of an article [23]. Second, it provides a curated data set in the JavaScript Object Notation format for experienced academic reviewers’ critical appraisal choices and decision-making methodologies. These data on the peer-review process provide a unique framework for modelling, quantifying, and ultimately automating the evidence quality assurance process and are unavailable elsewhere. Finally, due to the wide scope and depth of REDASA’s web crawling methodology, REDASA has already captured one of the world’s largest COVID-19–related data corpora for searches and curation. Our aim is to make these crucial data freely available and ensure that they are continuously updated to allow for rapid review and dissemination during and beyond the evolving pandemic.

For the long-term goal of conducting a semisupervised, live systematic review of data, several limitations and challenges need to be overcome. Our curation methodology resulted in a high turnover rate for the assessment of data. However, there was still variability in curator output, which was secondary to the variability in curators’ subjective critical appraisals. In this project, we relied on the prescreening of curators, which was conducted via academic portfolio screening and assessments for relevant literature review experience. This crucial quality control approach needs to be further developed to fully validate and enhance the accuracy of our curation methodology. A limitation of our preliminary data analysis was the qualitative, summative comparison of the EQUATOR checklist ratings to our quality ratings. This was due to the subcomponents of the used EQUATOR checklists, which did not use equal metrics for article quality, and the nonexhaustive quality criteria captured by these tools. Hence, future studies are needed to validate our quality ratings and identify a reliable metric for quality that is applicable across the academic and nonacademic literature captured by REDASA. In addition to ensuring the consistency of quality ratings, sustained curation work is required to ensure that the corpus includes greater numbers of studies across all designs and methodologies—specifically, RCTs (if available)—to ensure that the corpus is truly representative of data under examination.

Our framework has demonstrated proof-of-concept that by combining the discovery and ingestion pipeline, data lake repository, human curation platform, and NLP semantic search index of REDASA, it can provide curated responses to questions that are posed in natural language in the short term. In the long term however, based on the data insights that progressively validated the critical appraisal of our curation methodology, the ambition of REDASA is to conduct live systematic reviews by using semisupervised machine learning techniques to rapidly return high-quality, relevant evidence in response to queries for any discipline experiencing an “infodemic,” such as cancer or cardiovascular disease.

## Abbreviations

AGREE: Appraisal of Guidelines for Research and Evaluation
AWS: Amazon Web Service
CARE: Case Reports
CONSORT: Consolidated Standards of Reporting Trials
CORD-19: COVID-19 Open Research Dataset
EQUATOR: Enhancing the Quality and Transparency of Health Research
NER: named entity recognition
NLP: natural language processing
PRISMA: Preferred Reporting Items for Systematic Reviews and Meta-Analyses
RCT: randomized controlled trial
REDASA: Realtime Data Synthesis and Analysis
STROBE: Strengthening the Reporting of Observational Studies in Epidemiology
TRIPOD: Transparent Reporting of a Multivariable Prediction Model for Individual Prognosis or Diagnosis
